# Microbiological Characteristics and Surgical Management of Animal-Bite-Related Oral & Maxillofacial Injuries: A Single Center’s Experience

**DOI:** 10.3390/antibiotics10080998

**Published:** 2021-08-18

**Authors:** Johannes Spille, Juliane Schulz, Dorothee Cäcilia Spille, Hendrik Naujokat, Henning Wieker, Jörg Wiltfang, Aydin Gülses

**Affiliations:** 1 Department of Oral and Maxillofacial Surgery, Christian Albrechts University, UKSH, Campus Kiel, 24105 Kiel, Germany; Juliane.Schulz@uksh.de (J.S.); Hendrik.Naujokat@uksh.de (H.N.); Henning.Wieker@uksh.de (H.W.); Joerg.Wiltfang@uksh.de (J.W.); Aydin.Guelses@uksh.de (A.G.); 2 Department of Neurosurgery, University Hospital Münster, 48149 Münster, Germany; DorotheeCaecilia.Spille@ukmuenster.de

**Keywords:** animal bites, maxillofacial, treatment, antibiotics, hospitalization

## Abstract

The objective of the current study is to retrospectively evaluate animal-bite injuries and to gain insight into the epidemiology, accident consequences and treatment concept of these accidents in oral and maxillofacial surgery. Data of patients, who were admitted January 2015 and April 2021, were retrospectively evaluated regarding the patients’ characteristics (age, gender), facial distribution of substance defects/partial amputations, duration of hospitalization, operation treatments and antibiotic treatments. Data of 75 patients were included. Patients were bitten by dogs (*n* = 69.92%), cats (*n* = 4) and horses (*n* = 2). Lower eyelid/cheek complex was the most affected region (*n* = 37, 32.74%). Most of the patients between 0 and 3 years had to be operated on under general anesthesia (*p* = 0.011), while most of the adults could be operated on under local anesthesia (*p* = 0.007). In the age group 0–12 years, 30 patients (68%) were operated on under general anesthesia. Ampicillin/Sulbactam (48%) was the antibiotic most used. Antibiotics were adjusted after wound swabs in case of wound infections or critical wound conditions. This means that resistant antibiotics were stopped, and sensitive antibiotics were used. Structured surgical and antibiotic management of animal-bite wounds in the maxillofacial region is the most important factor for medical care to avoid long-term aesthetic consequences. Public health actions and policies under the leadership of an interdisciplinary committee could improve primary wound management, healing outcome and information status in the general population.

## 1. Introduction

Up to 30,000–50,000 injuries are associated with bite injuries every year in Germany, 60–80% resulting from dog-bite injuries [1]. Animal bites are not reportable in Germany, which is why there is no precise data on the frequency of bite injuries. However, animals live in more than a third of German households and are part of the everyday life of many citizens [2]. In addition, due to the steady increase in dog ownership, the current risk of being bitten by a dog during a lifetime is estimated to be over 50% [3]. After the upper and lower extremities, the face is the most common area for bite injuries [4]. These injuries often appear less serious but can lead to dangerous infections with tissue loss or even death. Especially in the face, these injuries can lead to disfiguring scars with lengthy treatments and aftercare. Moreover, the associated psychological burden on the patient is an important aspect, which is often neglected [5].

The infectious complications, which usually result from uncommon pathogens, are frequent and represent a special responsibility and medical challenge in oral and maxillofacial surgery. Normally, bite wounds are polymicrobial with a mixed flora of aerobic and anaerobic bacteria which can be difficult to control in medical treatment [6]. Furthermore, bites can trigger life-threatening diseases such as zoonotic infections—particularly rabies and tetanus [7]. The risk of animal bite wounds becoming infected can be as high as 50%, especially if wound management is inadequate [8].

A bite injury can be of many types: abrasions, puncture wounds, fractures, lacerations, tissue avulsions, etc. In each examination, it has to be kept in mind that the deep anatomical layers of the injured tissue may slide over each other during the bite and immediately back again, which can possibly lead to an underestimation of the true depth of the injury [9]. Moreover, animal bites can account for up to 1% of treatments in emergency departments and cause significant costs for the health system [10].

It is well known that bite injuries of the maxillofacial region are correlated with specific morbidity and mortality rates. Therefore, a clear understanding of the epidemiology, outcomes and therapeutic management options can permit better hospital care. The aim of the current study is to retrospectively evaluate the animal-bite-related maxillofacial injuries, to analyze the relation between the animal-bite injuries and the correspondent treatment management and to support as well as to standardize the current medical treatment methods. So far, there is no internationally recognized standard classification of bite injuries, although numerous methods describing bite injuries have been attempted [11]. That is the reason why communication between professionals in the field of bite injury treatment is difficult and an overview of the treatment options in relation to microbiological characteristics and surgical management is necessary.

## 2. Results

### 2.1. Demographic Data

A total of 69 patients treated for dog-bite injuries during the examination period were included (average age 19.22 ± 20.20 years, 43.48% male (*n* = 30) and 46.52% female patients (*n* = 39)). Distribution regarding the decades of lives is shown in Table 1. Furthermore, four patients were treated for cat-bite injuries (male = 1 (10 years and 11 months); female = 3 (3 years and 3 months, 16 years and 11 months, 24 years)) and two patients treated for horse-bite injuries (female = 2 (25 and 42 years)) were included.

**Figure 1:**
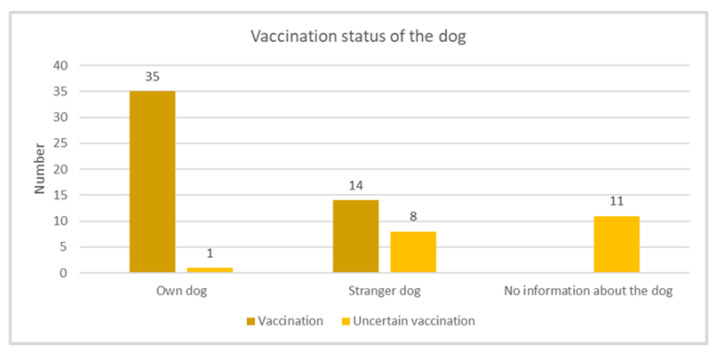
Schematic illustration of distribution regarding the vaccination status of dogs involved. Group 1 shows the vaccination status of the patients’ own dogs, group 2 the vaccination status of stranger dogs and group 3 shows the number of dogs that cannot be further assigned.

Of the bite injuries, 52.17% (*n* = 36) were caused by the patients’ own dogs, almost all of which were vaccinated (*n* = 35, 97.2%, *p* = 0.001). It so happens that 31.88% (*n* = 22) of the bite injuries were caused by stranger dogs, which were mostly vaccinated (*n* = 14, 63.63%). It is the case that 15.95% of the patients (*n* = 11) did not give any information about the dogs. This distribution is shown in Figure 1. In case of further doubts regarding the vaccination status of the animals, a vaccination against tetanus and rabies was carried out.

**Figure 2:**
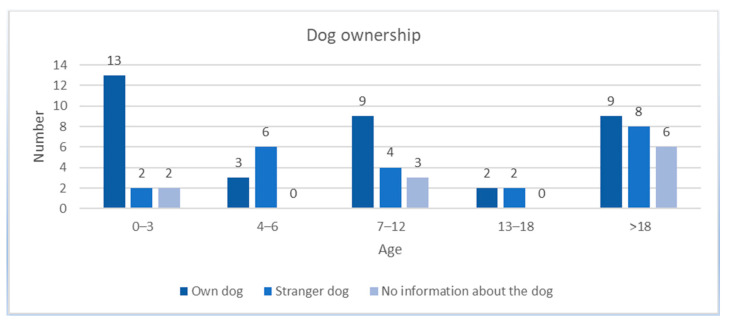
Distributions of the ages of the patients regarding dog ownership. The children between the ages of 0 and 3 were mostly involved, subject to bites from their own dog.

The distribution of the individual bite injuries in relation to the age of the patients is shown in Figure 2. Most patients between 0 and 3 years were bitten by their own dog (*n* = 13, 76.46%). This is a significant difference, considering the incidence of bite injuries in this age group—whether own dog or another dog—could be detected (*p* = 0.039). Two patients each (11.77%) were bitten by an unknown dog or no information about the dog was given. Six patients (66.67%) aged 4–6 years were bitten by a stranger dog and three (33.33%) by their own dog. In the 7–12 age group, nine (56.25%) were bitten by their own dog and four (25%) by a stranger dog. Three patients (18.75%) did not give any information about the dog. Of the 13–18-year-olds, two were bitten by their own dog and two by a stranger dog (each 50%). In the group of the adult patients (>18 years), nine (39.13%) were bitten by their own dogs and eight (34.78%) by a stranger dog. Six patients (26.09%) did not give any information about the dog.

Two of the patients with cat-bite injuries were bitten by their own cat. Two patients did not give any information about the cats (each 50%). No further anamnestic information was obtained on the horse-bite injuries.

### 2.2. Injury Patterns

Considering the number of substance defects and/or partial amputations, a total of 113 injury patterns were observed. The overall ratio of the number of injury patterns/patient was 1.51. The distribution of the affected injuries is related in Table 2: head (*n* = 2), upper eyelid/forehead (*n* = 13, 11.5%), lower eyelid/cheek (*n* = 37, 32.74%), ear (*n* = 3), nose (*n* = 5), upper lip (*n* = 18, 15.93%), lower lip (*n* = 12, 10.62%), chin (*n* = 4), neck (*n* = 3), hand (*n* = 3), wound infection/abscess (*n* = 13, 11.5%). Paresthesia of at least one facial region could be detected in four cases. No significant difference could be detected between the topography of the injury and the patient’s age. Regarding the CFI score, all injuries were declared to be complicated facial injuries.

### 2.3. Treatment and Hospitalization

**Figure 3:**
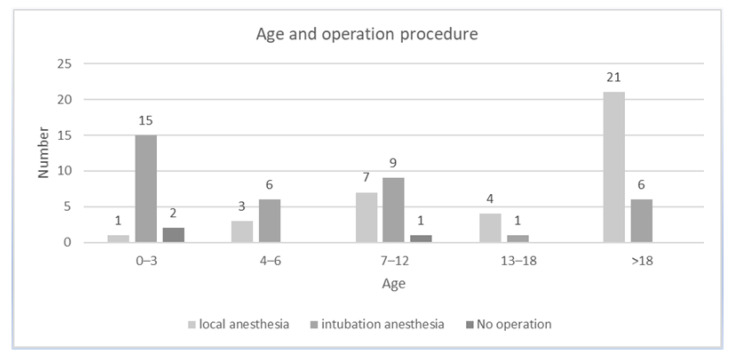
The assessment of the relationship between age and operative procedure shows that the management of bite-related injuries of patients between 0 and 3 years was mostly performed under general anesthesia.

In Figure 3, the ratio of patient’s age and the operation procedure is related. In the age group 0–3 years, 15 patients (83.33%) were operated on under general anesthesia. One patient could be operated on under local anesthesia and two were treated conservatively. In the 4–6-year-olds, six patients (66.67%) were treated under general anesthesia and three under local anesthesia (33.33%). In the group of 7–12-year-olds, nine patients (52.94%) were operated on under general anesthesia, seven (41.18%) under local anesthesia and one patient was treated conservatively. Of the 13–18-year-olds, four patients could be treated under local anesthesia and one patient had to be operated on under general anesthesia. In the group of the adult patients (>18 years), 21 patients (77.78%) were operated on under local anesthesia and six (22.22%) under general anesthesia. Descriptive analysis showed statistically significant differences regarding the incidence of surgical method in the groups 0–3 years (*p* = 0.011) and >18 years (*p* = 0.007).

There was no significant difference regarding the surgical procedure and antibiotic therapy. In the case of general anesthesia, 19 patients were treated with ampicillin/sulbactam, 16 patients with ampicillin/sulbactam/metronidazole and three patients with another combination. In the case of local anesthesia, 17 patients were treated with ampicillin/sulbactam, 14 patients with ampicillin/sulbactam/metronidazole and six patients with another combination.

**Figure 4:**
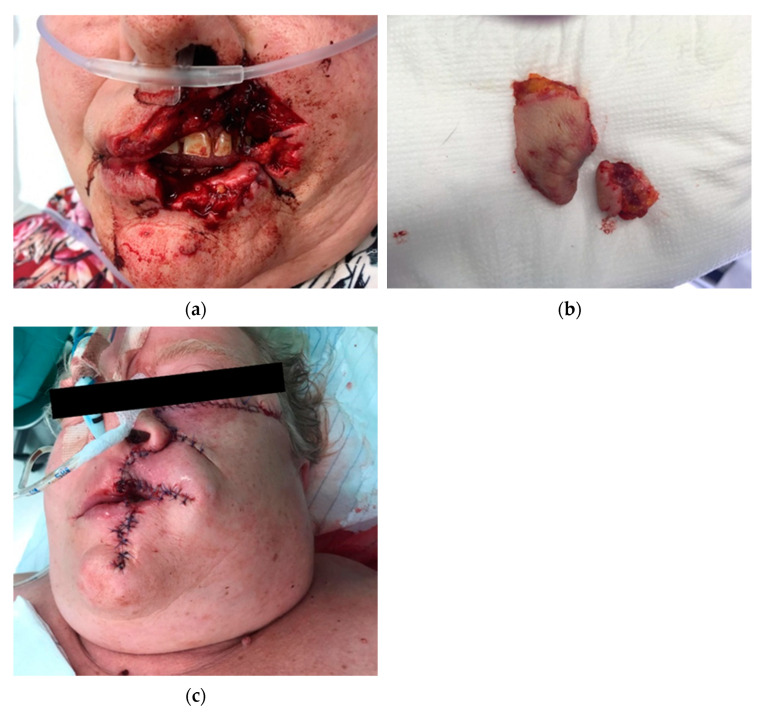
Initial wound defect secondary to a dog-bite injury of a 56-year-old woman by her own dog. The defect affects the upper lip up to the nose and a partial amputation of the upper lip is present (**a**). The involved tissue could not be re-transplanted (**b**); plastic reconstruction of the upper lip and mid-face soft tissue was performed by rotational flap under general anesthesia for the management of composite tissue defect (**c**).

**Figure 5:**
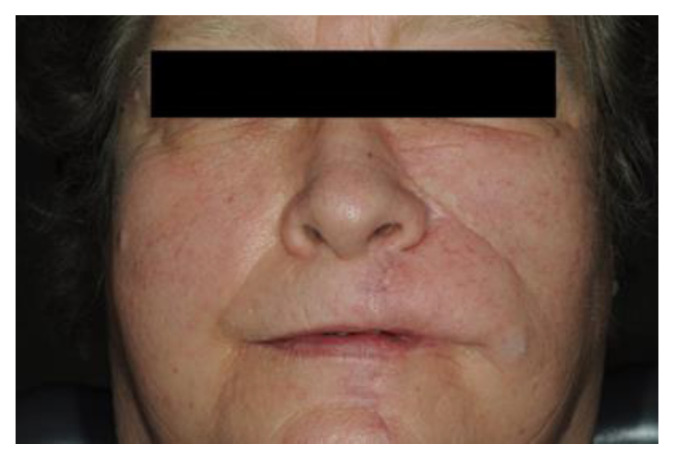
Frontal view of the patient reported in Figure 4 after six months. The patient underwent a commisuroplasty and a fat augmentation of the left upper lip for the management of the unilateral microstomia.

Figure 4 shows the extent that a dog-bite injury can have. A substance defect in the upper and lower lip was the result. Plastic reconstruction of the upper and lower lip was performed in an extensive operation under general anesthesia. Figure 5 shows the postoperative result for the patient about half a year after the accident.

**Figure 6:**
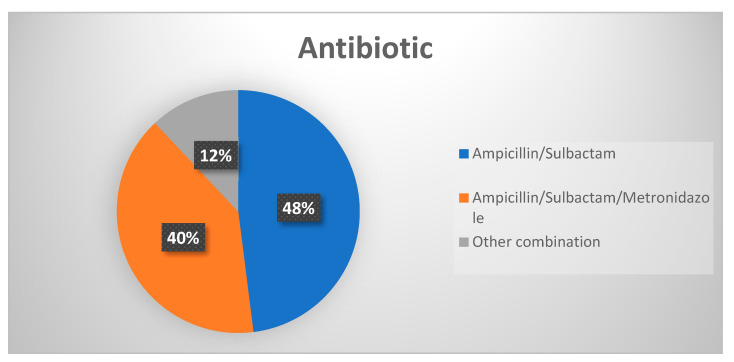
Distribution of antibiotic combinations. Listed are the two most used antibiotic combinations: ampicillin/sulbactam and ampicillin/sulbactam/metronidazole, and all other combinations as one group.

All patients were treated with an antibiotic during the hospitalization. The average hospital stay was 5.84 days (±2.81; max = 16; min = 1). Five patients left the clinic prematurely against medical advice. The immediate initial intravenous administration of the antibiotic was 5.25 days (±2.86; max = 15; min = 0). Furthermore, the administration of an oral antibiotic was continued as a follow-up treatment. The average of the administration was 6.13 days (±3.96; max = 21; min = 0). The antibiotic ampicillin/sulbactam was administered in 36 cases (48%), the combination of ampicillin/sulbactam and metronidazole in 30 cases (40%), and other antibiotic combinations were given in nine cases (12%), as shown in Figure 6. Other combinations were: cefuroxime/cefaclor, ampicillin/sulbactam/meropenem, ampicillin/sulbactam/moxifloxacin, ceftriaxone/metronidazole/clindamycin, cefotaxime/metronidazole, clindamycin/metronidazole, and ampicillin/sulbactam/metronidazole/moxifloxacin.

### 2.4. Pathogen Detection

Pathogen detection was carried out by 23 wounds (30.67%). The following pathogens were detected by microbiology: *Pasteurella multocida*, *Coagulase negative staphylococcus*, *Bergeyella zoohelcum*, *Capnocytophaga canimorsus*, *Escherichia coli*, *Bacteroides pyogenes*, *Candida albicans*, *Candida glabrata*, *Streptococcus pyogenes*, *Pasteurella canis*, *Propionibacterium acnes*, *Pseudomonas aeruginosa*, *Pasteurella stomatis*, *Sphingomonas paucimobilis*, *Streptococcus dysgalactiae* spp. equisimilis, *Corynebacterium* species, *Staphylococcus epidermidis*, *Neisseria* species, and *Bacillus* species. *Pasteurella* species was detected in most cases, with 47.8%. *Coagulase negative staphylococcus* was detected in 21.7%; the other pathogens were only detected in one to three cases.

## 3. Discussion

Bites from animals are relatively common and can lead to disfigurement, functional disability and infectious complications. Furthermore, wounds can require a debridement or repair in the operating room, which range from primary closure to microsurgical replantation, skin grafts, flaps or even facial transplantation. The international recognized CFI score displays a checklist regarding anatomical and functional classification of the injuries and describes bite injuries as well as facial or trigeminal nerve/salivary duct involvement, loss of tissues, gunshot wound, lachrymal drainage system and retrobulbar hematoma under soft tissue injuries as being complicated [12]. This can pose a challenge in medical care, thus making them an important public health problem. The annual treatments of animal-bite injuries generate costs in the range of millions of euros for both the patient and the entire population [9,13,14]. Therefore, there is a need for understanding of the cause and outcomes to determine local prevention and management strategies. To the best of our knowledge, the current study is the first analysis of animal-bite injuries in the maxillofacial region and their operative treatment and antibiotic management in the English literature for Germany.

Animal bites, especially dog bites, are counted among the most important etiological mechanisms of maxillofacial soft tissue injuries, being a global public health problem resulting in substantial costs to health care systems [15]. In the current study, 75 hospitalized patients presented with a total of 113 soft tissue injuries of the maxillofacial region. In the current study, most bite injuries are localized at the area of the lips, the periorbital region and the cheek. Piccart et al. confirmed a similar distribution in a 20-year review [16]. However, most patients were injured by dogs (*n* = 69). This might be explained by the number of pets, which are often dogs. This has also been previously stated by Park et al., who also declared that intensive contact with dogs is higher than with other pets [17].

Moreover, the described bite injuries are only a fraction of all bite injuries, as only those patients who required surgical or at least antibiotic treatment as inpatients were included. Other studies have found that the reporting rate for animal bites and the presentation and assessment of the wound in the hospital was exceptionally low, whereby immediate medical treatment often has the best medical outcome [18,19]. Thus, this study showed no significant results regarding surgical procedure and antibiotic therapeutics, suggesting that a particular therapy has no advantage, but immediate medical treatment is of the greatest importance. There is a great danger in trivialization of supposedly small wounds. However, these wounds require careful exploration, as fractures or deeper soft tissue damage may be hidden behind the apparent superficial injury. Thus, puncture wounds can be an increased risk for infection compared to other injury patterns [20]. This is likely to pose a high risk for wound infections and abscesses. Thus, 13 patients came to the hospital late, with serious inflammations, and had to be treated extensively. Antibiotic therapy was escalated after smear tests from the wound area during surgical procedure and after microbiological co-assessments. Ampicillin/sulbactam was administered as the standard initial antibiotic. Pfortmueller et al. confirmed the administration of β-lactam antibiotics as standard therapy; deviations with other preparations were only necessary in a few cases [3]. Furthermore, an individual antibiotic combination was also used in cases of wound healing disorders and/or exacerbation of wound healing. In particular, the combination of ampicillin/sulbactam and metronidazole was often found to be sensitive. In the case of penicillin allergy, alternatives such as clindamycin or cephalosporins can be used [21].

Antibiotic prophylaxis for animal bites is still to be debated; local wound care is described as the most important primary intervention measure [22]. Local wound disinfection and wound care were performed on all patients in the study. The recommendation is to irrigate several times with saline solution. Moreover, it is not necessary to use a solution containing iodine or antibiotics. Devitalized tissues should be removed according to the rules for debridement. Contaminants must be carefully removed [23]. In the facial area, primary wound closure is advisable due to the aesthetic consequences. A two-stage surgical wound closure is possible for wounds in other areas of the body and should always be discussed [24]. Additionally, anti-rabies vaccination should be administered when the vaccination status of the animal is unclear [25]. Different studies have recognized that prophylactic antibiotic therapy—except for bite wounds of the hand—did not achieve any advantage over placebo [26,27]. Other studies declared that an antibiotic therapy for facial bite wounds is necessary [28,29]. Preventive antibiotic therapy should also be considered for certain risk factors. For example, an antibiotic seems to make sense in the case of an accident event more than 9 h ago, in the case of pronounced wounds, heavy contamination or in immunosuppressed patients [30]. In the case of a pronounced bite injury, antibiotic therapy according to smear test and microbiological co-assessment should be established because it is superior to conservative therapy [22]. β-lactam antibiotics are the gold standard for antibiotic therapy against animal bites. Usually, a 7–14-days course is adequate for soft tissue infections; in the case of bone involvement, prolonged administration could be necessary [31]. Consequently, in the current study, the intravenous antibiotic infusion was 5.25 days. Furthermore, the administration of an oral antibiotic was continued as a follow-up treatment with an average of 6.13 days. Targeted antibiotic therapy is important to prevent the development of resistance. Improper use of antibiotics is presumably due to the time pressure in medical treatment, a lack of familiarity with guidelines and disagreement with guidelines explained [32].

Moreover, wounds are often infected after animal bites with a combination of aerobic and anaerobic bacteria and tend to present as polymicrobial [33,34]. The results of the current study supported this fact, such that each examined wound had different combinations of bacteria. Different studies show that the species of *Pasteurella* is the most common bacteria in animal-bite wounds [6,7]. Additionally, this could be supported in the current study in 47.8% of the cases with antimicrobial co-assessment. Other common bacteria include streptococci and staphylococci. Rasmussen et al. specified that every fifth dog bite becomes infected and can lead to major complications [35]. Therefore, the antibiotic treatment is often standard practice in clinics—as well as in this study—because of medical care regarding wound infection.

Thus, in the case of wound infection, surgical abscess incision and extensive surgical rehabilitation of the wound with debridement and using disinfectant were necessary. This complication presents the risk of unsatisfactory wound healing and pronounced scarring. Accordingly, the patients with an abscess had a larger wound and corrective surgery could be an option in the future. Therefore, it seems necessary that a doctor should assess the wound of the bite injury and decide in which setting the injury should be treated. In the end, most patients in this study had to be operated on; only three patients could be treated conservatively. The doctor decides whether an inpatient stay is necessary or whether the wound can be treated as an outpatient. Bhaumik et al. came to a similar conclusion that the best wound healing and outcome can only be achieved through professional treatment [36]. In this study, in most cases, satisfactory results could be achieved because of professional co-assessment of the wounds. Moreover, all patients were given a six-month follow-up appointment after hospitalization. If the patients were still dissatisfied with the outcome, a corrective surgery could then be planned.

Another important focus of the current study was the treatment of children because of the generated number of hospitalizations in the age group 0–12 years (*n* = 44, 58.67%). Kesting et al. detected that those children are the most common victims of animal bites, particularly of dog bites [37]. The most common site of injury in children was the face, especially the middle third. The size of children, the relation of the head to the body, their careless handling of the animal, familiarity with the animal at play and limited motor skills to provide defense are believed to account for this [25,38]. However, there were no significant differences in the topography of bite injuries and the age range in this study. In all, 30 of the 44 children (68.2%) had to be operated on under general anesthesia. As well as the trauma of the animal bite, the operation under general anesthesia with a subsequent longer hospital stay could leave irreversible damage on children’s physiological and psychological development. Moreover, children do not understand animal-bite attacks and may subsequently have considerable fear at the sight of animals [39].

Children may experience nightmares, flashbacks, social isolation, anxiety, phobias, low self-esteem or negative body image issues. For this reason, Shen et al. even recommend subsequent psychological treatment for every animal-bite injury to minimize long-term psychological damage [40]. In addition, the parents or brothers and sisters of the injured child may be plagued by feelings of guilt, especially if the bite was caused by their own pet [41].

Besides that, children belong to the risk group of life-threatening injuries in the facial area because the cranial bone is still relatively soft. An injury to the brain or inflammation in the brain area can have fatal consequences. Antibiotic therapy is always indicated in such a pattern of injury [42].

The current study also has some major limitations. Since the study was based on the retrospective analysis of clinical records, data regarding details such as the dog breed, dog owner or the postoperative outcome were not always available. Besides that, patients included in the study were limited to those who were mostly injured severely enough to require hospital care. The patients who were injured but did not stay in hospital at a university clinic could not be included. Furthermore, the application of a standard classification to evaluate the severity of animal-bite injuries was not used in the initial wound inspection of the doctors until now. In the future, a large database or a prospective study for animal-bite-related maxillofacial injuries should be established. Moreover, a standard classification evaluation of animal-bite injuries should be established in every clinic to obtain better understanding for the treatment of these wounds.

## 4. Materials and Methods

This retrospective study was undertaken at Universitätsklinikum Schleswig-Holstein (UKSH) Campus Kiel, Kiel-Germany. Ethical approval was obtained from the Ethics Commission of the Faculty of Medicine at the Christian-Albrechts-University, Kiel (D502/21).

UKSH is the only trauma center which provides treatment for oral and cranio-maxillofacial injuries around the clock, 365 days a year. Data of the patients with animal-related bite injuries presented at the clinic of Department of Oral and Maxillofacial Surgery between January 2015 and April 2021 were collected via ORBIS Information-Management-System (ORBIS AG, Saarbrücken, Germany). All patients who stated “animal-bite injury” in the database fields for “cause of injury” and those who were treated in hospital were considered. Incomplete patient charts and patients who received outpatient treatment were excluded. In addition, patients who did not give informed consent were excluded from the study. The consent of underage patients was given by their parents.

The data included:

- Patient characteristics (age, gender);
- Own animal (±, if documented);
- Vaccine of the animal (±, if documented);
- Injury characteristics (area and extent of facial injury);
- Concomitant injuries (extremities, abdomen, thorax, etc.);
- Management;
- Pathogen;
- Duration of hospitalization;
- Antibiotic therapy (intravenous and oral);
- Duration of antibiotic therapy.

Bite injuries were assessed according to the international recognized and standardized comprehensive facial injury (CFI) score. All patients were treated according to the same regimen. First, a wound inspection was carried out by a doctor, followed by further imaging diagnostics (sonography, computer tomography, magnetic resonance imaging), if required. In the case of serious injuries, the attending doctor ordered imaging diagnostics to quantify the extent of the injuries in depth, to quantify an abscess, to exclude fractures and to detect or exclude foreign bodies. After that, the patients were operated on (local/general anesthesia) or treated conservatively. All patients received antibiotic therapy as inpatients due to the severity of the injury. If necessary, the antibiotic therapy was continued orally on an outpatient basis. All patients received a near-term follow-up appointment and a follow-up appointment six months after the accident.

### Statistical Analysis

Statistical analyses were performed using SPSS. A descriptive analysis of the patient’s characteristics was performed. Categorical variables were presented as number and percentage. Normally distributed and non-normally distributed continuous variables were expressed as mean (±SD) or median (range). Categorical data were presented as total counts and percentages. The ratio of the extent of the injury and duration of hospitalization as well as the duration of antibiotic treatment were calculated. The relation between variables was evaluated by chi-square test, Fisher exact test or Mann–Whitney U test. Associations were considered significant when the *p* value was <0.05.

## 5. Conclusions

This study showed the extent of the effect which animal bites, especially dog bites, can have. This incidence is probably an underestimation of the real number of animal-related injuries because only the hospitalized injuries were detected. The injuries can cause severe physical injury and aesthetic compromise as a result, especially by trivialization and not seeing a doctor. Thus, awareness among the general population and especially parents regarding more cautious and prudent attitudes towards animals could reduce the amount and the severity of animal-related injuries. Public health actions and policies under the leadership of an interdisciplinary committee (physicians, veterinarians, microbiologists, wound managers and others) could improve primary wound management, healing outcome and information status in the general population.

## Figures and Tables

**Table 1:** Distributions of dog-bite injuries in relation to the age groups. Group 1 shows the 0–3-years-old, group 2 shows the 4–7-years-old, group 3 shows the 8–12-years-old, group 4 shows the 13–18-years-old and group 5 shows the >18-years-old.

Decade of Life	*n*	%
0–3	18	24
4–6	9	12
7–12	17	22.67
13–18	5	6.67
>18	26	34.67

**Table 2:** Distribution of substance defects/partial amputations depending on the body parts of the oral and maxillofacial region.

Specific Body Part	Number of Injuries
Head	2
Upper eyelid/forehead	13
Lower eyelid/cheek	37
Ear	3
Nose	5
Upper lip	18
Lower lip	12
Chin	4
Neck	3
Hand	3
Wound infection/abscess	13

## Data Availability

The data is available in this manuscript.
